# Report of a Case of Scrofulous Caries of the Head, the Thigh Bone, in an Adult, Cured by Codliver Oil and Iodine Potassium

**Published:** 1855-07

**Authors:** J. R. Mills

**Affiliations:** Huntington, Indiana


ART. V.—*Report of a case of Scrofulous Caries of the Head, the Thigh Bone, in an Adult, Cured by Codliver Oil and Iodine Potassium.* By J. R. Mills, M. D., Huntington, Indiana.

*Professor Davis :—Dear Sir :*—The following case is one in which I have felt a great interest, and I submit it to your disposal :

Mr. P. Wire, aged 23, scrofulous diathesis, was attacked about the 1st of February last with pneumonia, for which he was treated by Drs. H. and W., of Lagro, for some two weeks, at which time the pneumonic symptoms subsided ; during this time he had suffered some pain in the hip and knee, but of not such severity as to attract much attention until about this time, when it seemed to be the whole difficulty. But the attending physicians considered it of no moment or consequence, and Dr. Sheets, a Uriscopean, and Leyman, an Alopathist, (the two constituting a firm in this place for the convenience and accommodation of people of different views,) were called to take charge of the case. There diagnosis was, " weakness of the nerves," their prognosis unhesitatingly favorable. After these professional ceremonies, and a few remarks on the indications of the urine, the patient was put upon the use of quinine and rhubarb—3 grs. of the former with 6 of the latter every three hours, with mustard to the knee. Such was the treatment for 5 or 6 weeks. During this time they never looked at the hip, in fact never saw it. At this time, 3d of April, I saw the patient, found the history of the case to be that this pain had come on during his first sickness, as before stated, and treated as before, with a gradual increase of weakness His general appearance indicated great prostration, with profuse night sweats, hectic flush of the cheek, diarrhœa. The diseased limb, flexed and proped up and bound to the opposite one, to prevent the shaking of the limb as the family walked over the floor,—could not bear the least motion of the knee, or the least pressure upon the Trocanter Major, in the direction of the Acetabulum without extreme suffering ; while lying still, felt as much pain in the knee as in the hip. I found the Nates on the effected side drawn up, and the lame limb about ¾ of an inch the longer.

Such, in brief, is the condition in which I found the patient on the 3d of April last.

The diagnosis was plain; that of scrofulous caries of the thigh bone,—it was at least my diagnosis. I am aware that it is a nice point to diagnose correctly in hip affections, particularly between this disease and chronic ulcerations of the cartilage.

My prognosis was not difficult, for it has always appeared to me, that to diagnose scrofulous caries, was virtually prognosticating the death of the limb, if not the patient, yet I will admit that Drs. may be mistaken,—such was the case in this instance, so far as prognosis was concerned.

I first directed my treatment for the profuse night sweats and diarrhœa, for some three days, when I put in a large seton, gave him Codliver oil in teaspoonful doses every 6 hours, and Iodide Potassium 3 grs. every 6 hours in solution—diet, beef or chicken tea, with perfect rest.

April 12th.—I found him much the same, with less disposition to sweat,—continued treatment.

April 20th.—Patient much the same, except, perhaps, some better appetite, diarrhœa stopped, some night sweats yet. Continued treatment.

April 30th.—I think some little improvement. The patient does not complain when they walk the floor, can move the ankle a little without pain, appetite better, night sweats better. Continued treatment.

May 9th.—I found him this morning evidently better in every particular, can straighten the well leg and support the other alone, and can give the knee some lateral motion, seton acts well, in fine spirits. Continued treatment.

May 20th.—Patient much better, was lifted into a chair yesterday, and sat up some time, every symptom improving.

May 30th.—Still improving, sitting up when helped out. Continued treatment.

June 6th.—Patient hopping about the chouse on crutches, with his leg flexed so the toes just touch the floor.

June 11th.—Patient still improving—out in the yard to-day—no pain in the hip, except the attempt to bear his weight upon the limb. I have left him on the use of the oil. The limb will always be crooked.

Huntington, June 12th, 1855.

